# Lipoprotein(a) is a Promising Residual Risk Factor for Long-Term Clinical Prognosis in Peripheral Arterial Disease

**DOI:** 10.3400/avd.oa.22-00046

**Published:** 2022-09-25

**Authors:** Kimimasa Sakata, Hisao Kumakura, Ryuichi Funada, Yae Matsuo, Kuniki Nakashima, Toshiya Iwasaki, Shuichi Ichikawa

**Affiliations:** 1 Department of Cardiovascular Surgery, Cardiovascular Hospital of Central Japan (Kitakanto Cardiovascular Hospital), Shibukawa, Gunma, Japan; 2 Department of Cardiovascular Medicine, Cardiovascular Hospital of Central Japan (Kitakanto Cardiovascular Hospital), Shibukawa, Gunma, Japan

**Keywords:** lipoprotein(a), all-cause mortality, major adverse cardiovascular event, peripheral arterial disease, limb events

## Abstract

**Objectives:** We investigated the relationship between plasma lipoprotein(a) [Lp(a)] level and long-term prognosis, cardiovascular events, or pure leg events (LE) in patients with peripheral arterial disease (PAD).

**Materials and Methods:** We prospectively enrolled 1104 PAD patients. The endpoints were LE, cerebrovascular- or cardiovascular-related death (CVRD), all-cause death (ACD), and major adverse cardiovascular events (MACE).

**Results:** The incidences of LE, CVRD, ACD, and MACE were correlated with Lp(a) level (P<0.05). Lp(a) was positively correlated with low-density lipoprotein cholesterol and C-reactive protein (CRP) and negatively correlated with estimated glomerular filtration rate (eGFR). In the Cox multivariate regression analysis, high Lp(a), CRP, age, low ankle-brachial pressure index (ABI), eGFR, albumin, critical limb ischemia (CLI), cerebrovascular disease (CVD), and diabetes were associated with LE; high Lp(a), age, CRP, low ABI, body mass index, eGFR, albumin, CLI, coronary heart disease (CHD), CVD, and diabetes were associated with CVRD; high Lp(a), CRP, age, low ABI, eGFR, albumin, CLI, and CVD were associated with ACD; and high Lp(a), CRP, age, low eGFR, albumin, CLI, CHD, and diabetes were associated with MACE (P<0.05). Statins improved all endpoints (P<0.01).

**Conclusion:** Lp(a) was a significant residual risk factor for LE, CVRD, ACD, and MACE in PAD patients.

## Introduction

Lipoprotein(a) [Lp(a)] is a complex polymorphic lipoprotein composed of a low-density lipoprotein particle and glycoprotein apo(a) through apoB-100.^1)^ Lp(a) shares high-level homology with plasminogen, and Lp(a) activates monocytes and the migration of macrophage foam cells into the arterial wall.^1,2)^ The plasma Lp(a) level is correlated with calcification of coronary artery and coronary heart events.^2,3)^ Several reports have demonstrated that Lp(a) is an independent risk factor for coronary heart disease (CHD) and cerebrovascular disease (CVD).^1–3)^

Lipid abnormality is a predominant risk factor for both cardiovascular events and clinical prognosis. Thus, lipid-lowering treatment is effective in improving the incidence of CHD or CVD. Several studies have reported that statins exert an antiatherogenic effect on CHD and reduce cardiovascular events.^4)^ However, cardiovascular risks persist despite intensive lipid-lowering treatment with statins.^5)^ Several additional factors have been proposed to be the mechanisms underlying these residual risks in cardiovascular diseases.^5,6)^ Plasma Lp(a) is a causal residual risk factor correlated with cardiovascular events.^6)^ The correlation is continuous without a threshold and is not affected by the levels of low-density lipoprotein cholesterol (LDL-C).^7)^ High plasma Lp(a) levels increase the incidence of peripheral arterial disease (PAD) and PAD-related hospitalization.^8,9)^ The condition of patients with PAD is complicated by severe atherosclerosis, which leads to a high rate of cardiovascular events and mortality.^10,11)^ Furthermore, we have reported that the higher serum level of Lp(a) is associated with increased risks of mitral or aortic valve stenosis and CHD in PAD patients.^3,12)^ Plasma Lp(a) may be a predictor of atherosclerosis correlated with cardiovascular events, including lower-limb events in PAD patients.

However, the role of Lp(a) as the residual risk factor for long-term survival, cardiovascular events, and fate of the limb based on the serum Lp(a) level has not been clearly confirmed in PAD patients. This research aimed to investigate the long-term clinical prognosis and cardiovascular and/or limb events according to the Lp(a) levels in patients with PAD.

## Materials and Methods

### Patients

PAD patients were recruited in the observational cohort study at our hospital between April 2001 and September 2021. The design and methods of the study complied with the principles of the Declaration of Helsinki, and the detailed methods were approved by the ethics committee of our institution (CCJ-EA-006). All subjects provided written informed consent for this study. The study objectives and characteristics of the subjects were symptoms with intermittent claudication or critical limb ischemia (CLI), an ankle-brachial pressure index (ABI)<0.90, and a femoro-popliteal or iliac artery stenotic lesion≥70% detected via ultrasound or angiography.

### Primary clinical analysis for patients

The primary clinical data included the baseline analysis for age, smoking history, body mass index (BMI), ABI, hypertension, and diabetes mellitus (DM). The basic clinical data included serum albumin, glucose, creatinine, LDL-C, high-density lipoprotein cholesterol, total cholesterol, triglyceride, D-dimer, and C-reactive protein (CRP) levels of a morning blood sample. Moreover, we collected a fasting sample for the Lp(a) measure. Measurement of the serum Lp(a) was performed via a turbidimetric immunoassay.

### Follow-up and endpoints

The medical status and vital signs of each patient were assessed at 4- or 6-month intervals based on questionnaires and hospital data administered at the Foot Care Club in our institution.^13,14)^ Ischemic cerebral disease was determined as a new neurological defect and a focal lesion identified via computed tomography or magnetic resonance imaging. The criteria of myocardial infarction were previously defined.^13,15)^ Limb events were defined as above-the-ankle amputation and progression of a new stenosis or restenosis identified as ≥50% stenosis and falling in the ABI of ≥0.15 via angiography or ultrasonography in the peripheral arterial lesions.

The endpoints were pure leg events (LE: presence of a new peripheral lesion, repeat revascularization for a peripheral artery, or major amputation), cerebrovascular- or cardiovascular-related death (CVRD), all-cause death (ACD), and major adverse cardiovascular events (MACE: ACD, non-fatal CVD, transient ischemic attack, or non-fatal myocardial infarction).

### Statistical methods

Quantitative variables were expressed as median (interquartile range), and categorical variables were expressed as frequencies. Quantitative variables were compared using the Mann–Whitney U test and categorical variables using the chi-squared test. Cumulative event rates were defined through the Kaplan–Meier analysis for LE, CVRD, ACD, and MACE in the follow-up period. In the Cox regression analysis, the levels of Lp(a) were log-transformed to ameliorate distributions as predictive risk variables. In the Cox univariate model, the hazard ratios (HR) and 95% confidence intervals (CI) were analyzed for individual valuables. Significant factors in these analyses were calculated to determine the efficient risk factors associated with these outcomes in the multivariate regression model. P values <0.05 were considered significant. All data were calculated using SPSS Statistics version 25.0 (IBM Corp., Armonk, NY, USA).

## Results

### Characteristics of the subjects and causes of death

A total of 1163 subjects were enrolled into this cohort. Among the enrolled subjects, 1104 completed the follow-up. The median and mean ages were 74 (68–79) and 73.5±9.9 years, respectively. The median follow-up period was 68 (25–117) months. There were 581 deaths (52.6%). The causes of death were cardiovascular related (n=216, 37.2%), malignancy (n=122, 21.0%), pneumonia (n=87, 15.0%), cerebrovascular related (n=72, 12.4%), and others (n=84, 14.5%).

The median and mean Lp(a) levels were 21.0 (11.6–35.0) and 27.4±23.6 mg/dL. Thus, the subjects were divided into two groups on the base of the Lp(a) levels with median: L1, ≤21.0 mg/dL (n=563); L2, ≥21.1 mg/dL (n=541). The clinical characteristics of patients with PAD divided by median into two groups are summarized in **Table 1**. Patients with higher Lp(a) had lower BMI and estimated glomerular filtration rate (eGFR), serum albumin, and triglyceride levels as well as higher LDL-C, CRP, and D-dimer levels. The prevalence of CLI was higher, and treatment with an angiotensin receptor blocker was higher in rates in the L2 category.

**Table 1:** Table 1 Characteristics of subjects in all patients, L1, and L2 based on lipoprotein(a) levels

	All patients n=1104	L1 Lp(a)≤21.0 mg/dL n=563 (51.0%)	L2 Lp(a)≥21.1 mg/dL n=541 (49.0%)	P-value
Age (year)	73 (67–79)	73 (66–79)	74 (67–81)	0.012
Male sex	834 (75.5%)	436 (77.4%)	398 (73.6%)	0.142
Body mass index (kg/m^2^)	22.0 (19.8–24.3)	22.4 (20.3–24.5)	21.5 (19.1–24.1)	<0.001
Critical limb ischemia	206 (18.7%)	91 (16.2%)	115 (21.3%)	0.031
Intermittent claudication	898 (81.3%)	472 (83.8%)	426 (78.7%)	0.031
ABI	0.69 (0.50–0.81)	0.70 (0.52–0.81)	0.67 (0.49–0.80)	0.061
Cerebral infarction	205 (18.6%)	105 (18.7%)	100 (18.5%)	1.000
Coronary heart disease	389 (35.2%)	187 (33.2%)	202 (37.3%)	0.166
Hypertension	738 (66.8%)	375 (66.6%)	363 (67.1%)	0.898
Diabetes mellitus	443 (40.1%)	232 (41.2%)	211 (39.0%)	0.462
Smoking	808 (73.2%)	416 (73.9%)	392 (72.5%)	0.634
Medications				
Statin	794 (71.9%)	398 (70.7%)	396 (73.2%)	0.354
Aspirin	704 (63.8%)	351 (62.2%)	353 (65.2%)	0.315
Thienopyridines	392 (35.5%)	196 (34.8%)	196 (36.2%)	0.660
Cilostazol	279 (25.3%)	133 (23.6%)	146 (27.0%)	0.213
Beraprost	414 (37.5%)	208 (36.9%)	206 (38.1%)	0.709
β-blocker	164 (14.9%)	82 (14.6%)	82 (15.2%)	0.800
ARB	346 (31.3%)	177 (31.4%)	169 (31.2%)	0.948
Ca antagonist	583 (52.8%)	293 (52.0%)	290 (53.6%)	0.630
Revascularization	645 (58.4%)	330 (58.6%)	315 (58.2%)	0.903
Basic metabolic panel				
Lipoprotein(a) (mg/dL)	21.0 (11.6–35.0)	12.0 (7.1–16.1)	35.0 (27.0–51.0)	<0.001
Serum albumin (g/dL)	4.0 (3.7–4.2)	4.0 (3.8–4.2)	3.9 (3.7–4.2)	0.001
eGFR (mL/min/1.73 m^2^)	56.2 (42.3–68.6)	57.9 (45.7–72.4)	52.8 (38.8–65.7)	<0.001
CRP (mg/dL)	0.19 (0.08–0.50)	0.16 (0.08–0.40)	0.20 (0.09–0.59)	0.002
D-dimer (µg/dL)	0.9 (0.5–2.0)	0.8 (0.5–1.6)	1.1 (0.6–2.3)	<0.001
Total cholesterol (mg/dL)	186 (160–214)	185 (160–212)	187 (161–218)	0.264
LDL-C (mg/dL)	113 (90–135)	110 (87–132)	117 (94–136)	0.006
Triglyceride (mg/dL)	124 (87–172)	129 (92–179)	119 (81–163)	0.001
HDL-C (mg/dL)	48 (39–58)	48 (39–58)	49 (40–59)	0.522

ABI: ankle-brachial pressure index; ARB: angiotensin receptor blocker; eGFR: estimated glomerular filtration rate; CRP: C-reactive protein; LDL-C: low-density lipoprotein cholesterol; HDL-C: high-density lipoprotein cholesterol

### Correlational statistics among Lp(a) and cardiovascular risk factors

In the simple Pearson correlation analysis, Lp(a) was significantly positively correlated with LDL-C, total cholesterol, and CHD and negatively correlated with ABI, BMI, and eGFR (P<0.05). The correlational statistics among Lp(a) and these significant risk factors were analyzed via a stepwise forward multiple regression analysis (**Table 2**). The plasma Lp(a) levels had significant positive correlations with LDL-C and CRP and negative correlations with eGFR (P<0.05).

**Table 2:** Table 2 Correlational statistics between lipoprotein(a) level and other risk factors

Factor	β	B	95%CI	P-value
LDL-C (mg/dL)	0.195	0.137	0.091 to 0.183	<0.001
C-reactive protein (mg/dL)	0.082	1.153	0.247 to 2.059	0.013
eGFR (mL/min/1.73 m^2^)	−0.076	−0.070	−0.130 to −0.010	0.022

R^2^=0.045, F for change in R^2^=13.909, P<0.001 β: standardized coefficient; B: non-standardized coefficient; CI: confidence interval for B; LDL-C: low-density lipoprotein cholesterol; eGFR: estimated glomerular filtration rate

### LE, CVRD, ACD, and MACE

**Figure 1:**
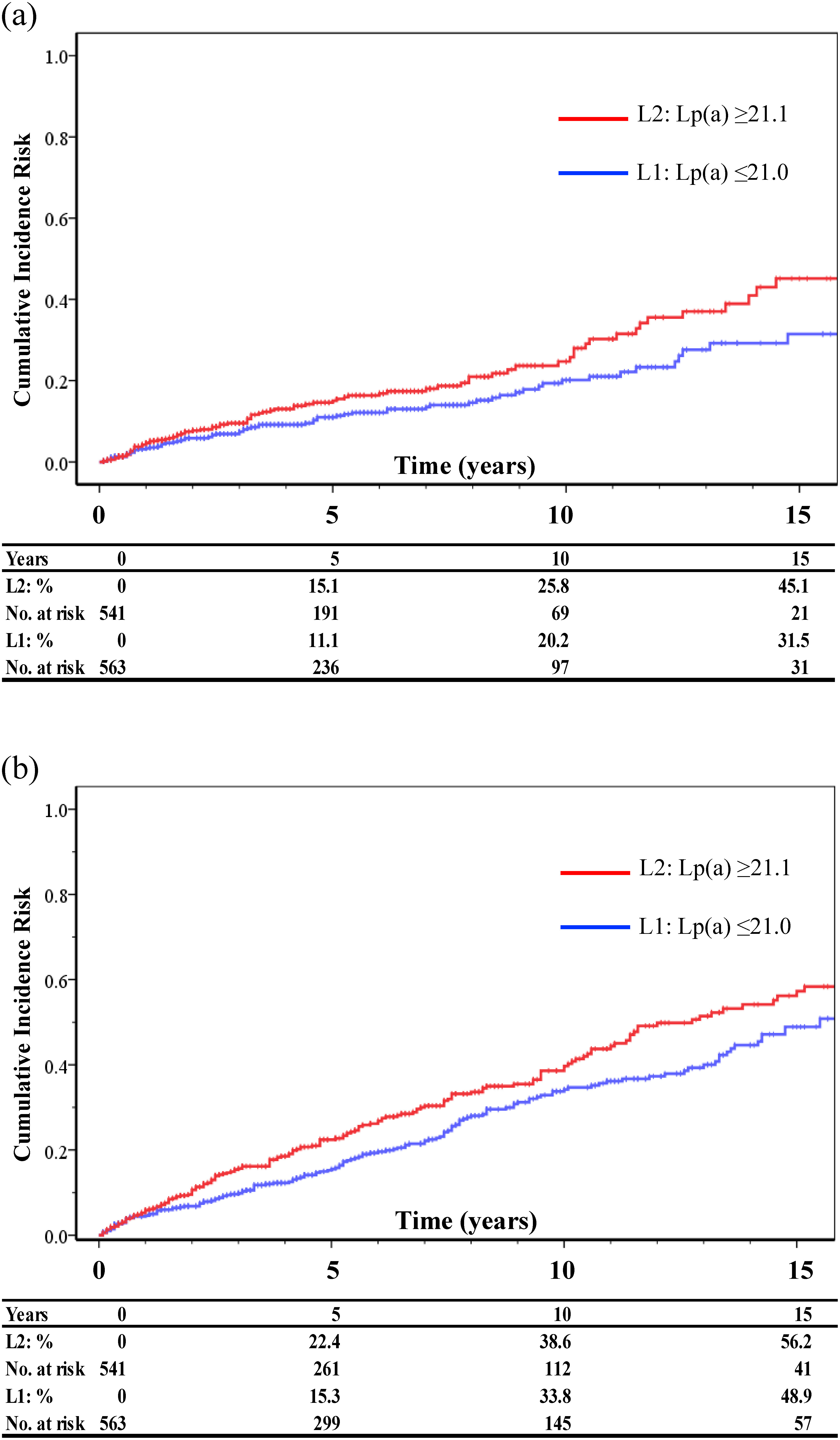
Fig. 1 (**a**) Cumulative incidence of pure leg events (LE) according to lipoprotein(a) levels is demonstrated with significant difference between L1 and L2 (P=0.038). (**b**) Cumulative incidence of cardiovascular- or cerebrovascular-related death (CVRD) according to lipoprotein(a) levels is demonstrated with significant difference between L1 and L2 (P=0.015).

**Figure 1a** presents the cumulative incidences of the 5-, 10-, and 15-year rates for LE between L1 and L2, and the incidence of LE was significantly higher in L2 than in L1 (P=0.038). In the Cox univariate regression analysis, high Lp(a), age, CRP, D-dimer, low eGFR, ABI, BMI, albumin, CLI, DM, and CVD were significantly associated with LE. Treatment with statins improved LE (P<0.05). In Cox multivariate regression analysis, high Lp(a), age, CRP, low eGFR, ABI, albumin, CLI, DM, and CVD were associated with LE. Treatment with statins was found to reduce LE (**Table 3**, P<0.05).

**Table 3:** Table 3 Cox multivariate regression analysis for pure leg events (LE), cardiovascular-related death (CVRD), all-cause death (ACD), and major adverse cardiovascular events (MACE)

	LE			CVRD			ACD			MACE		
	Multivariate analysis			Multivariate analysis			Multivariate analysis			Multivariate analysis		
	HR	95%CI	HR	HR	95%CI	p-value	HR	95%CI	p-value	HR	95%CI	p-value
Age (year)	1.025	1.016–1.035	<0.001	1.053	1.037–1.068	<0.001	1.055	1.043–1.067	<0.001	1.037	1.026–1.047	<0.001
ABI	0.656	0.472–0.911	0.012	0.465	0.398–0.611	0.001	0.548	0.336–0.894	0.016	0.739	0.527–1.034	0.078
Critical limb ischemia	1.290	1.003–1.638	0.047	1.413	1.003–1.991	0.048	1.432	1.090–1.882	0.010	1.485	1.164–1.896	0.001
Cerebral infarction	1.273	1.033–1.569	0.024	1.823	1.367–2.430	<0.001	1.300	1.028–1.644	0.028	1.067	0.850–1.339	0.478
Coronary heart disease				1.317	1.007–1.723	0.044	1.224	0.992–1.511	0.060	1.659	1.373–2.005	<0.001
Diabetes mellitus	1.339	1.121–1.599	0.001	1.406	1.073–1.841	0.013	1.182	0.966–1.446	0.104	1.367	1.133–1.651	0.001
Lipoprotein(a) (mg/dL)	1.115	1.010–1.231	0.030	1.183	1.015–1.380	0.032	1.129	1.025–1.398	0.044	1.117	1.008–1.239	0.035
eGFR (mL/min/1.73 m^2^)	0.994	0.990–0.998	0.002	0.990	0.984–0.996	0.001	0.984	0.978–0.991	<0.001	0.994	0.991–0.998	0.007
Serum albumin (g/dL)	0.626	0.512–0.765	<0.001	0.556	0.407–0.760	<0.001	0.588	0.430–0.803	<0.001	0.617	0.497–0.765	<0.001
CRP (mg/dL)	1.102	1.053–1.153	<0.001	1.120	1.045–1.200	0.001	1.112	1.036–1.194	0.003	1.135	1.079–1.194	<0.001
Statin	0.509	0.423–0.613	<0.001	0.449	0.334–0.605	<0.001	0.463	0.342–0.667	<0.001	0.425	0.347–0.520	<0.001

HR: hazard ratio; CI: confidence interval; eGFR: estimated glomerular filtration rate; ABI: ankle-brachial pressure index; CRP: C-reactive protein

**Figure 1b** presents the cumulative incidences of the 5-, 10-, and 15-year rates for CVRD between L1 and L2, and the incidence of CVRD was significantly higher in L2 than in L1 (P=0.015). In the Cox univariate analysis, high Lp(a), CRP, age, D-dimer, low ABI, eGFR, albumin, CLI, DM, CHD, and CVD were related to CVRD. Treatment with statins or aspirin and revascularization were associated with CVRD (P<0.05). In the multivariate model, high Lp(a), CRP, age, low ABI, eGFR, albumin, CLI, DM, CHD, and CVD were also related with CVRD, and treatment with statins decreased CVRD (**Table 3**, P<0.05).

**Figure 2:**
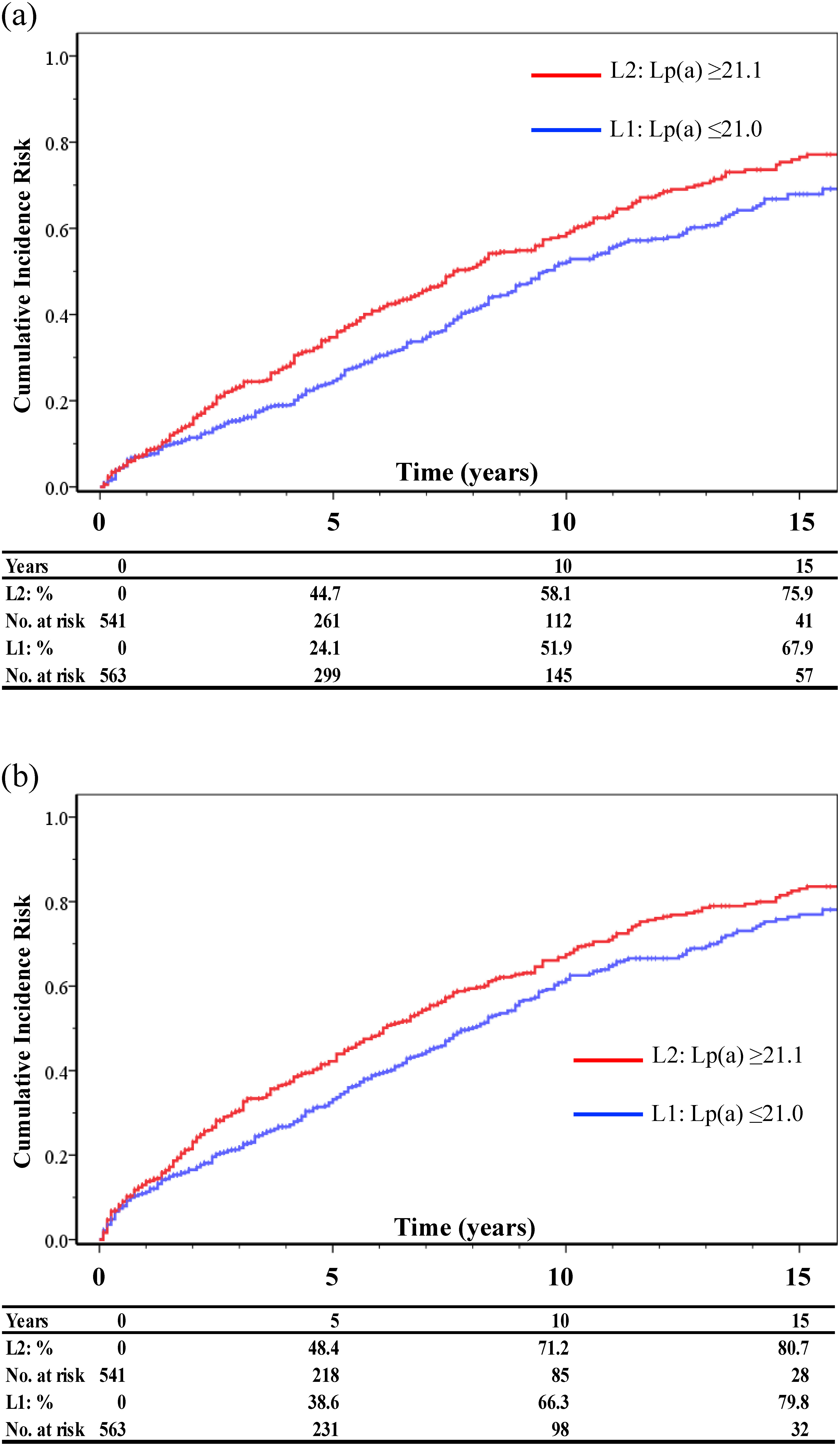
Fig. 2 (**a**) Cumulative incidence of all-cause death (ACD) according to lipoprotein(a) levels is demonstrated with significant difference between L1 and L2 (P=0.002). (**b**) Cumulative incidence of major adverse cardiovascular events (MACE) according to lipoprotein(a) levels is demonstrated with significant difference between L1 and L2 (P=0.003).

**Figure 2a** presents the cumulative incidences of the 5-, 10-, and 15-year rates for ACD between L1 and L2, and the incidence of ACD was significantly higher in L2 than in L1 (P=0.002). In the Cox univariate analysis, high Lp(a), age, CRP, D-dimer, low eGFR, ABI, BMI, albumin, CLI, DM, CHD, and CVD were significantly associated with ACD. Treatment with statins and revascularization were associated with mortality (P<0.05, respectively). In the multivariate model, high Lp(a), age, CRP, low eGFR, ABI, albumin, CLI, and CVD were associated with ACD. Treatment with statins was associated with ACD (**Table 3**, P<0.05).

**Figure 2b** presents the cumulative incidences of MACE between L1 and L2, and the incidence of MACE was significantly higher in L2 than in L1 (P=0.003). In the Cox univariate regression analysis, high Lp(a), CRP, age, D-dimer, low eGFR, ABI, albumin, CLI, DM, CHD, and CVD were associated with MACE. Statin was related to these events (P<0.05, respectively). In the Cox multivariate regression analysis, high Lp(a), age, CRP, low eGFR, albumin, CLI, CHD, and DM were associated with MACE, and treatment with statins improved MACE (**Table 3**, P<0.05).

## Discussion

The current study provided the first 15-year clinical evidence for the relationships between plasma Lp(a) level and life expectancy, cardiovascular events, or lower-limb events in PAD patients. In this analysis, the cumulative incidences of CVRD and ACD were significantly increased relative to the higher plasma Lp(a) category. High plasma Lp(a) levels are related to coronary artery calcification and CHD.^2,3,16)^ We have reported that the serum Lp(a) level was correlated with mitral or aortic valve stenosis and CHD in PAD patients.^3,12)^ Single-nucleotide polymorphisms (SNPs) in Lp(a) gene (rs10455872) are also related to aortic valvular calcification and stenosis.^17)^ An inverse correlation was reported between the Lp(a) concentrations and apo(a) isoform size.^18)^ Furthermore, low-molecular-weight apo(a) phenotypes significantly increase the risk of PAD.^19)^ In the current study, higher plasma Lp(a) levels were significantly related to LE. Higher plasma Lp(a) levels increase the incidences of PAD and PAD-related hospitalization.^8,9)^ Thus, the concentrations of Lp(a), low-molecular-weight apo(a) phenotypes, and SNPs in the rs10455872 were associated with the progression of PAD, CHD, and aortic valve stenosis.^6,16,18)^

ACD was associated with higher plasma Lp(a) and CRP levels and age as well as lower eGFR level. Despite earlier studies reporting that Lp(a) concentrations are mainly fixed by genetic factors, recent studies have demonstrated that Lp(a) is induced by chronic inflammation.^20)^ In the current study, the plasma Lp(a) levels were significantly positively correlated with CRP levels. Higher CRP is a significant predictor for LE, CVRD, ACD, and MACE in PAD patients.^10,14)^ Several studies have demonstrated that Lp(a) is enhanced by the immune system.^20,21)^ Especially, several immune diseases, including Crohn’s disease and rheumatoid arthritis, are related to elevated serum levels of Lp(a) that increase the incidence of CVD and CHD.^20)^ In these patients, inflammatory responses induced by cytokines may affect both atherosclerosis development and cardiovascular remodeling.^22,23)^ Moreover, Lp(a) induces monocyte chemoattractant protein, tumor necrosis factor alpha (TNF-α), and interleukin (IL)-1β in macrophages.^24)^ High TNF-α levels are related to peripheral arterial restenosis, cardiac dysfunction, and MACE.^22,23)^ Lp(a) has a high-level homology with plasminogen and activates monocytes and the migration of the macrophage foam cells into the arterial wall.^1,2,24)^ The Lp(a) molecule is related to platelet aggregation and vascular remodeling mediated by inflammation with these cells.^24)^ Meanwhile, studies have reported that high IL-6 levels enforce Lp(a) synthesis.^25)^ IL-6 blockade with monoclonal antibodies suppresses Lp(a) synthesis and apolipoprotein (a) expression.^20)^ Thus, inflammatory responses increase cytokine and Lp(a) levels that increase cardiovascular risks in these subjects.

Several studies have demonstrated that the abnormal threshold levels of Lp(a) are ≥30–50 mg/dL.^7,9,16)^ In the current study, the threshold Lp(a) level of ≥30 or 50 mg/dL did not confirm significant clinical outcomes, whereas the Lp(a) level with a median of 21.0 mg/dL showed significant differences in all outcomes. Suwa et al. have reported that the Lp(a) level with a median of 21.5 mg/dL showed significant difference on long-term coronary events in subjects receiving statin after coronary intervention.^26)^ The correlation between plasma Lp(a) level and clinical prognosis is continuous without a threshold and is not affected by the LDL-C level.^7)^ Because elevated plasma Lp(a) levels occur at birth, patients may be affected by the cardiovascular risk beginning very juvenile in life.^27)^

In this study, the plasma Lp(a) levels had a significant negative correlation with eGFR. Several studies have reported that the kidney has a function in plasma Lp(a) catabolism. Thus, plasma Lp(a) levels are elevated in patients with low eGFR and chronic kidney disease with large apo(a) isoforms.^28)^ PAD patients frequently have low eGFR, which is a significant risk factor for CHD, CVD, and LE.^10,11)^ High plasma Lp(a) levels were also a significant predictor for all outcomes in the current study. These results indicated that patients with high Lp(a) levels enhanced by low eGFR have severe systemic atherosclerosis related to high LE and CVRD incidence.

Several clinical trials have reported that statins reduce cardiovascular events.^4)^ In this study, treatment with statins was also effective for improving LE, CVRD, ACD, and MACE. However, higher plasma Lp(a) level persisted as a significant risk factor for all outcomes adjusted by statin therapy, eGFR, and other risk factors. Statins do not decrease plasma Lp(a) levels.^29)^ Studies have demonstrated that proprotein convertase subtilisin/kexin type 9 inhibitors decrease serum Lp(a) and LDL-C levels and significantly decrease the risk of cardiovascular event or major adverse limb events.^30)^ Thus, plasma Lp(a) levels may be a causal residual risk factor correlated with long-term survival, cardiovascular events, and fate of the limb in patients with PAD.

### Limitations

This study has several limitations: 1) the current outcomes were analyzed using data from a single institution; 2) the number of subjects was relatively small; 3) the prescription rate of statins during the entire follow-up period was relatively lower when comparing the current study with the guidelines; and 4) changes in lifestyle and health condition during follow-up time were not evaluated in the current study. These issues need to be investigated in further prospective studies with a large number of subjects for the current outcomes in patients with PAD.

## Conclusion

The cumulative incidences of LE, CVRD, ACD, and MACE were intensified relative to higher plasma Lp(a) levels. Plasma Lp(a) was identified as a promising residual predictor for LE, CVRD, ACD, and MACE in patients with PAD.
